# Factors Associated with Major PICU Interventions in Adolescents Hospitalized for Intentional Pharmaceutical Poisoning

**DOI:** 10.3390/children13081058

**Published:** 2026-08-08

**Authors:** Ebru Guney Sahin, Cansu Durak

**Affiliations:** Department of Pediatric Intensive Care, Sancaktepe Sehit Prof. Dr. Ilhan Varank Training and Research Hospital, University of Health Sciences, Istanbul 34785, Türkiye; bzmrt@hotmail.com

**Keywords:** adolescent, self-poisoning, intentional pharmaceutical poisoning, pediatric intensive care unit, poisoning severity, suicide attempt, toxicology

## Abstract

**Highlights:**

**What are the main findings?**

- Among 125 adolescents admitted to the PICU for intentional pharmaceutical poisoning, 15 (12.0%) required major PICU interventions, including mechanical ventilation, vasoactive support, CRRT, TPE, or intensive neurological/cardiovascular support.
- Lower Glasgow Coma Scale scores, higher lactate levels, Poisoning Severity Score (PSS) ≥ 3, and ingestion of medications belonging to family members were associated with the need for major PICU interventions, whereas mortality was rare (0.8%).

**What are the implications of the main findings?**

- Major PICU interventions may represent a more clinically meaningful measure of poisoning severity than mortality alone in adolescents with intentional pharmaceutical poisoning.
- Safe household medication storage may represent an important family-based strategy to reduce the risk of severe intentional pharmaceutical poisoning.

**Abstract:**

**Objective:** Intentional pharmaceutical poisoning is one of the most common methods of self-harm among adolescents and frequently results in pediatric intensive care unit (PICU) admission because of the potential for severe pharmaceutical-related toxicity. Although mortality is generally low, a subset of patients may require advanced intensive care interventions. **Methods:** This retrospective observational cohort study was conducted in the pediatric intensive care unit of a tertiary referral hospital between January 2022 and January 2026 and included adolescents admitted to the PICU due to intentional pharmaceutical poisoning. Demographic characteristics, psychiatric history, clinical findings, laboratory parameters, administered treatments, and clinical outcomes were evaluated. The primary outcome measure was defined as the requirement for a major PICU intervention, including invasive or noninvasive mechanical ventilation, vasoactive support, continuous renal replacement therapy (CRRT), therapeutic plasma exchange, extracorporeal membrane oxygenation (ECMO), intensive care-level seizure management, or clinically significant arrhythmia requiring intensive care support. Patients with and without major PICU intervention requirements were compared. **Results:** A total of 125 adolescent patients were included in the study, and the majority were female. Multiple-pharmaceutical ingestion and a history of psychiatric diagnoses were common comorbidities. Although the clinical course was stable in most patients, 15 patients (12%) required major pediatric intensive care interventions. Patients requiring major interventions had lower Glasgow Coma Scale scores, higher lactate levels, more frequent symptomatic presentation, and higher rates of Poisoning Severity Score (PSS) ≥ 3. Overall mortality in the cohort was low. **Conclusions:** Although most adolescents admitted to the PICU due to intentional pharmaceutical poisoning experienced mild-to-moderate toxicity, a subgroup required advanced intensive care support. Neurological impairment at presentation, symptomatic clinical presentation, and markers of increased physiologic stress appeared to be associated with more severe clinical courses. In pediatric poisonings, evaluating the need for advanced intensive care support rather than focusing solely on mortality may better reflect clinical severity.

## 1. Introduction

Intentional pharmaceutical ingestion is one of the most common methods of self-harm during adolescence and represents an increasingly important public health problem. In recent years, a marked increase in hospital admissions related to intentional overdose and self-poisoning has been reported, with this increase appearing particularly prominent among adolescent girls [1,2]. Due to psychosocial stressors, the rising prevalence of psychiatric diagnosis, and easy access to medications, intentional pharmaceutical poisonings have become a frequently encountered clinical condition in pediatric emergency departments and pediatric intensive care units (PICUs).

Although most adolescent pharmaceutical poisonings follow a mild-to-moderate clinical course, some patients may develop severe neurological, cardiovascular, or metabolic toxicity requiring advanced intensive care support [3,4]. Altered mental status, neurological impairment, hemodynamic instability, and metabolic disturbances have particularly been reported to be associated with more severe clinical outcomes [5].

Most pediatric studies on intentional pharmaceutical poisoning have primarily focused on epidemiological characteristics, ingested pharmaceutical classes, or mortality. However, factors associated with major PICU interventions among adolescents admitted to the PICU have been less well characterized. Previous multicenter studies have shown that many poisoned patients admitted to the PICU ultimately do not require advanced intensive care support [3,6], highlighting the need to better identify patients at risk of requiring major intensive care interventions.

In this study, we aimed to evaluate the demographic, clinical, and laboratory characteristics of adolescents admitted to the PICU following intentional pharmaceutical poisoning and to identify factors associated with major PICU interventions.

## 2. Materials and Methods

### 2.1. Study Design and Setting

This retrospective single-center observational cohort study included adolescents admitted to the pediatric intensive care unit due to intentional pharmaceutical poisoning between January 2022 and January 2026.

The study was approved by the Scientific Research Ethics Committee of Sancaktepe Sehit Prof. Dr. Ilhan Varank Training and Research Hospital (Approval No: E-46059653-050.04-306694309; approval date: 25 February 2026). The study was conducted in accordance with the principles of the Declaration of Helsinki. Due to the retrospective study design and the use of anonymized patient data, the requirement for informed consent was waived by the ethics committee.

### 2.2. Patient Selection

Adolescents aged 12–18 years who were admitted to the PICU following intentional pharmaceutical ingestion during the study period were included. Only patients with self-harm intent were eligible for inclusion; those presenting after pharmaceutical poisoning for recreational purposes were not included. Patients with unintentional accidental poisonings, environmental toxin exposures, corrosive substance ingestions, chronic pharmaceutical toxicities, admissions related solely to alcohol or recreational substance use, and patients with incomplete clinical data were excluded.

### 2.3. PICU Admission Criteria

The decision for PICU admission was based on the patient’s clinical condition at presentation, the characteristics of the ingested pharmaceutical(s), the anticipated risk of delayed toxicity, the need for neurological or hemodynamic monitoring, and the clinical judgment of the attending pediatric intensivist. In addition, recommendations from the National Poison Information Center (NPIC; Ulusal Zehir Danışma Merkezi, 114), which is routinely consulted in the management of pediatric poisoning cases in Türkiye, constituted an integral part of the admission process. Patients for whom NPIC recommended intensive care monitoring were generally admitted to the PICU in conjunction with the attending pediatric intensivist’s clinical assessment. No standardized institutional admission protocol for intentional pharmaceutical poisonings was available during the study period; therefore, admission decisions were individualized according to these clinical considerations.

Pediatric Risk of Mortality III (PRISM III) scores were calculated using the worst physiological values obtained within the first 24 h following PICU admission [7].

### 2.4. Data Collection

Demographic characteristics, presenting clinical findings, laboratory parameters, ingested medication classes, psychiatric history, administered treatments, and clinical outcomes were retrospectively obtained from the electronic medical record system.

Recorded clinical variables included Glasgow Coma Scale (GCS) score, symptomatic presentation, somnolence/confusion, agitation, seizure, hypotension, arrhythmia, and respiratory distress. Symptomatic poisoning was defined as the presence of any clinical sign or symptom considered attributable to poisoning at hospital presentation. Laboratory evaluation included blood gas parameters, pH, bicarbonate, base excess, and lactate levels.

The presence of multi-pharmaceutical ingestion, history of psychiatric diagnosis, previous suicide attempt, and alcohol or recreational substance use was also evaluated. Time from ingestion to hospital admission was defined as the interval between pharmaceutical ingestion and hospital presentation. Analyses involving time-to-presentation variables were performed using available-case analysis due to missing data in some patients. No imputation method was applied for missing data. Medication source was classified as either the patient’s own prescribed medication or medications belonging to family members available within the household.

Psychotropic medication ingestion was defined as ingestion involving antidepressants, antipsychotics, anxiolytic/sedative agents, mood stabilizers, and methylphenidate-containing medications.

### 2.5. Outcome Measures

The primary outcome measure was defined as the requirement for “major PICU interventions.” Major PICU interventions were defined as advanced organ support therapies or severe clinical events requiring intensive care-level monitoring and management. This composite endpoint was considered present if at least one of the following conditions occurred:

- Invasive or noninvasive mechanical ventilation requirement, including intubation performed for airway protection due to low Glasgow Coma Scale scores.
- Vasoactive support requirement.
- Continuous renal replacement therapy (CRRT).
- Therapeutic plasma exchange (TPE).
- Extracorporeal membrane oxygenation (ECMO).
- Seizures requiring intensive care-level neurological monitoring or treatment.
- Clinically significant arrhythmias requiring continuous cardiac monitoring, antiarrhythmic therapy or intensive care support.

Secondary outcomes included Poisoning Severity Score (PSS), antidote requirement, PICU length of stay, and mortality.

PSS was retrospectively assessed according to the European Association of Poison Centres and Clinical Toxicologists (EAPCCT) criteria based on the maximum clinical toxicity observed during hospitalization [8].

### 2.6. Statistical Analysis

Statistical analyses were performed using IBM SPSS Statistics software version 22.0 (IBM Corp., Armonk, NY, USA). The distribution of continuous variables was assessed using the Kolmogorov–Smirnov test. Since most continuous variables were not normally distributed, data were presented as median and interquartile range (IQR). Categorical variables were expressed as numbers and percentages.

Comparisons between categorical variables were performed using the chi-square test or Fisher’s exact test, as appropriate. Continuous variables were compared using the Mann–Whitney U test.

Receiver operating characteristic (ROC) curve analysis was performed to evaluate the discriminative performance of admission lactate levels for predicting major PICU interventions. The area under the curve (AUC), sensitivity, specificity, and 95% confidence intervals (CIs) were calculated.

Analyses involving time-to-presentation variables were performed using available-case analysis due to missing data in some patients. A two-sided *p* value of <0.05 was considered statistically significant.

Due to the limited number of patients requiring major PICU interventions, multivariable analysis was not performed.

## 3. Results

A total of 125 adolescents admitted to the PICU due to intentional pharmaceutical poisoning were included in the study. The median age of the patients was 16.2 years (IQR: 15.0–16.9), and 88.0% of the cohort consisted of female patients. Evaluation of clinical findings at presentation demonstrated a median GCS score of 15.0 (IQR: 15.0–15.0). Multi-pharmaceutical ingestion was observed in 63.2% of patients. A concomitant psychiatric diagnosis was present in 49.6% of patients, while 23.2% had a history of previous suicide attempts. The median time from ingestion to hospital admission was 2 h (IQR: 1.0–4.25 h) (Table 1).

The most commonly involved medication classes were antidepressants (36.8%), analgesics (32.8%), and antipsychotics (31.2%). Other less commonly involved agents included antibiotics, methylphenidate, antihypertensive agents, antiepileptic medication, antihistamines, iron preparations, antidiabetic agents, antiarrhythmic agents, and colchicine (Table 2).

Symptomatic poisoning was present in 64.8% of patients. The most common symptom was nausea/vomiting (33.6%), followed by dizziness (22.4%), somnolence/confusion (17.6%), and agitation (11.2%). Hypotension (6.4%), tachycardia (4.8%), arrhythmia (4.0%), seizure (2.4%), and respiratory distress (2.4%) were observed less frequently. According to the PSS classification, 36.0% of patients were categorized as PSS 0, 43.2% as PSS 1, 8.8% as PSS 2, 11.2% as PSS 3, and 0.8% as PSS 4 (Supplementary Table S1).

Regarding treatment modalities, gastric lavage (36.8%) and activated charcoal administration (36.0%) were the most commonly applied supportive therapies. Antidote therapy was administered in 20.8% of patients. The requirement for invasive or noninvasive mechanical ventilation was 3.2%, vasoactive support was required in 4.8%, and CRRT was required in 4.0% of patients. Therapeutic plasma exchange was performed in two patients (1.6%), while no patient required ECMO (Table 3).

Major PICU interventions were observed in 15 patients (12.0%). There was no significant difference in age between patients with and without major PICU interventions [16.7 (15.8–17.1) vs. 16.0 (15.0–16.9) years, *p* = 0.356]. Time from ingestion to hospital admission was also similar between groups [2.5 (1.0–7.5) vs. 2.0 (1.0–4.75) hours, *p* = 0.566]. The proportion of patients with PRISM III scores > 0 was significantly higher in the major intervention group than in the no major intervention group (20.0% vs. 1.8%, *p* = 0.012) (Table 4). Because time-to-presentation data were unavailable for 35 patients, a sensitivity analysis comparing patients with complete and missing data was performed. No significant differences were observed in overall clinical severity, Poisoning Severity Score, major PICU intervention requirement, or mortality, suggesting that missingness was unlikely to have materially influenced these findings.

GCS score < 15 at presentation was more frequent among patients requiring major PICU interventions (33.3% vs. 12.7%), although this difference did not reach statistical significance (*p* = 0.053). Lactate levels were significantly higher in the major intervention group [1.72 (1.04–3.80) vs. 1.17 (0.91–1.50), *p* = 0.010]. Patients requiring major interventions had significantly longer PICU stays [3.0 (3.0–4.0) vs. 2.0 (1.0–2.0) days, *p* < 0.001] (Table 4).

Among the 15 patients requiring major PICU interventions, multi-pharmaceutical ingestion was present in 12 (80.0%) patients. Colchicine-containing ingestions accounted for three cases (20.0%), antihypertensive agents were involved in five cases (33.3%), and antiepileptic drugs in three cases (20.0%). Most patients requiring major interventions had ingested combinations of multiple drug classes, and no single drug class predominated consistently.

Female sex distribution was similar between groups (93.3% vs. 87.3%, *p* = 0.434). Although multi-pharmaceutical ingestion was more common in the major intervention group, this difference did not reach statistical significance (80.0% vs. 61.5%, *p* = 0.162). Use of medications belonging to family members was significantly more frequent in the major intervention group (80.0% vs. 50.0%, *p* = 0.026). In contrast, psychiatric diagnoses were observed less frequently in the major intervention group (20.0% vs. 54.6%, *p* = 0.012). Although previous suicide attempts were less common in the major intervention group, this difference did not reach statistical significance (6.7% vs. 25.9%, *p* = 0.085). No significant difference was observed between groups regarding substance/alcohol use (Table 4).

Symptomatic presentation was significantly more frequent in the major intervention group (93.3% vs. 60.9%, *p* = 0.010). Antidote administration rates were similar between groups (26.7% vs. 20.0%, *p* = 0.551). The proportion of patients with PSS ≥ 3 was significantly higher in the major intervention group (93.3% vs. 0.9%, *p* < 0.001) (Table 4).

Variables with sparse cell counts (particularly previous suicide attempt, substance/alcohol use, and PSS ≥ 3) should be interpreted cautiously because of the limited number of patients requiring major PICU interventions. Odds ratios are presented as unadjusted effect estimates. *p* values are derived from the corresponding univariate group comparisons (Pearson’s χ^2^ test, Fisher’s exact test, or Mann–Whitney U test, as appropriate).

Odds ratios were not reported for PSS ≥ 3 because sparse cell counts resulted in an unstable estimate (quasi-complete separation).

**Figure 1:**
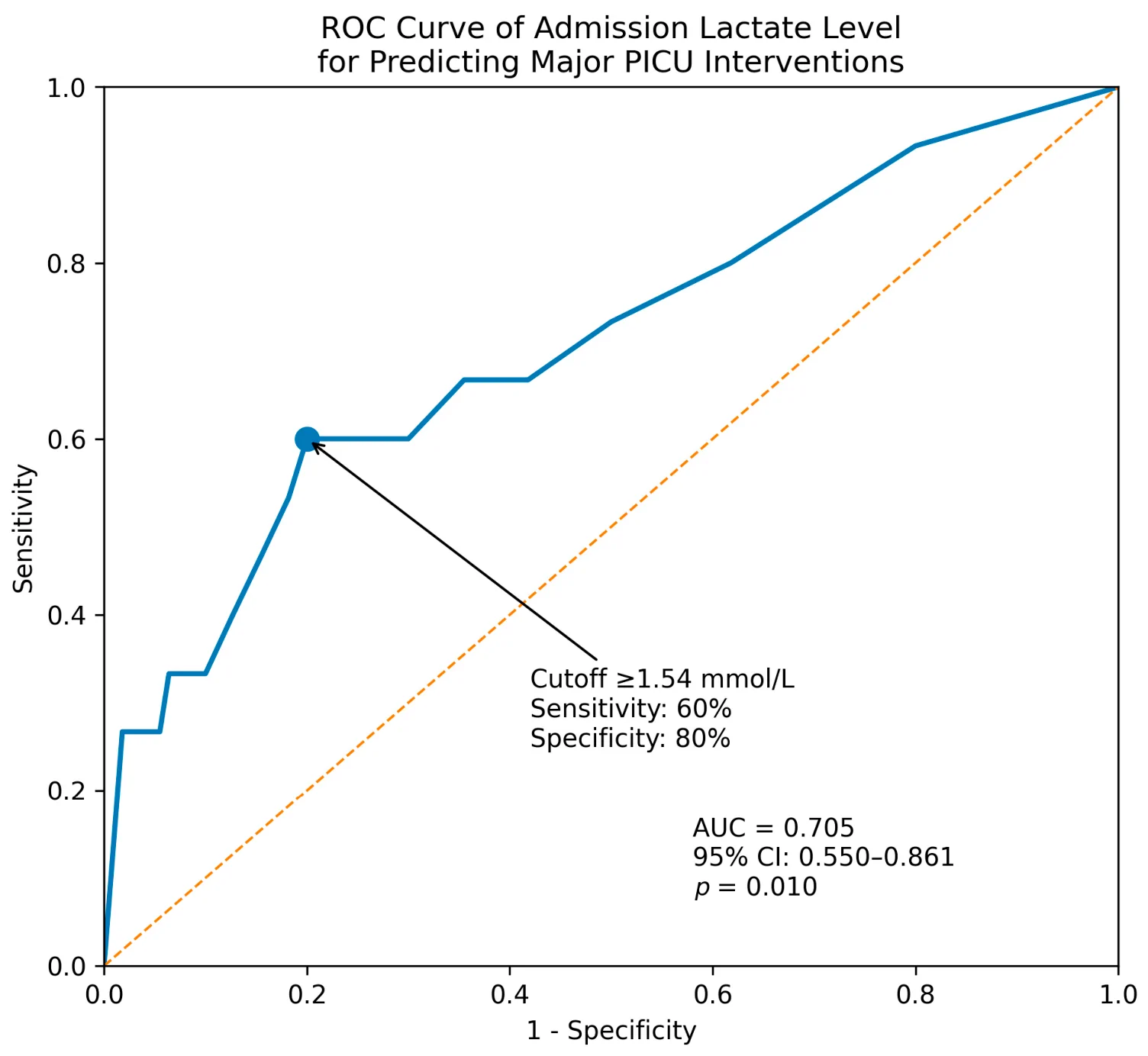
Receiver operating characteristic (ROC) curve analysis of admission lactate level for predicting major PICU interventions in adolescents with intentional pharmaceutical poisoning. The area under the curve (AUC) was 0.705 (95% CI: 0.550–0.861, *p* = 0.010). A lactate cut-off value of 1.54 mmol/L demonstrated 60% sensitivity and 80% specificity. The dotted diagonal line represents the line of no discrimination (AUC = 0.5).

ROC curve analysis demonstrated moderate discriminative performance of admission lactate level for identifying patients requiring major PICU interventions (AUC: 0.705, 95% CI: 0.550–0.861, *p* = 0.010) (Figure 1). A lactate cut-off value of 1.54 mmol/L was associated with a sensitivity of 60% and specificity of 80%.

When asymptomatic and symptomatic poisoning groups were compared, symptomatic patients were younger [16.0 (15.0–16.9) vs. 16.4 (15.4–16.9) years, *p* = 0.024]. No significant differences were observed between groups regarding female sex distribution, multi-pharmaceutical ingestion, history of previous suicide attempts, Glasgow Coma Scale score, lactate levels, or pH values. The rates of PSS ≥ 3 (17.3% vs. 2.3%, *p* = 0.010) and requirement for major PICU interventions (17.3% vs. 2.3%, *p* = 0.010) were significantly higher in the symptomatic group (Supplementary Table S2).

## 4. Discussion

In this retrospective cohort study, the majority of adolescents admitted to the PICU due to intentional pharmaceutical poisoning presented with mild-to-moderate toxicity and generally favorable clinical outcomes. Nevertheless, approximately 12% of patients required major PICU interventions such as mechanical ventilation, vasoactive support, CRRT, or TPE. Despite the very low mortality rate observed, a clinically significant subgroup developed severe physiological deterioration requiring organ support. These findings suggest that mortality alone may underestimate disease severity in intentional pharmaceutical poisoning. Similar observations have been reported in previous cohorts, where severe poisoning required mechanical ventilation, vasoactive support, or extracorporeal therapies despite low mortality [3,4,9,10,11]. Accordingly, composite outcomes incorporating major PICU interventions may better characterize clinically relevant disease severity.

Although the requirement for major PICU interventions was relatively low, the high proportion of patients classified as PSS 0 was notable. This suggests that PICU admission decisions are influenced not only by current symptom severity but also by ingestion of high-risk agents, the possibility of delayed toxicity, the need for close monitoring, and psychiatric evaluation. Previous studies likewise reported that many poisoned children admitted to the PICU ultimately required no intensive care-level intervention [3,6]. Similarly, in an emergency department cohort, 68% of children with pharmaceutical exposure required no specific therapy and only 4.6% were hospitalized [12], indicating that treatment intensity does not necessarily correspond to the level of care.

Patients requiring major PICU interventions in our study demonstrated lower GCS scores, higher lactate levels, and more frequent symptomatic presentations, suggesting more pronounced physiological impairment at hospital admission. In contrast, time from ingestion to hospital admission was not associated with the requirement for major PICU interventions, indicating that clinical severity may be more closely related to the degree of physiological deterioration at presentation rather than exposure duration alone. Previous studies have similarly reported that neurological impairment, altered mental status, and early physiological deterioration are associated with more severe toxicity [5,13,14]. In addition, ROC analysis demonstrated moderate discriminative performance of admission lactate level for identifying patients requiring major PICU interventions. The proposed cut-off value of 1.54 mmol/L should be considered exploratory and interpreted as a tool for early risk stratification rather than a definitive clinical threshold. Given the limited number of outcome events in our cohort, this cut-off requires external validation in larger cohorts before it can be considered clinically applicable. The relatively modest lactate elevations observed in our cohort are more likely to reflect nonspecific physiological stress or early tissue hypoperfusion associated with severe pharmaceutical poisoning than toxin-specific metabolic effects.

Approximately two-thirds of patients ingested multiple pharmaceuticals, comparable to the 47.2–66.0% reported in recent adolescent intentional poisoning cohorts [11,15,16,17]. Antidepressants, analgesics, and antipsychotics were the most frequently involved agents, consistent with previous pediatric studies [2,18,19,20]. Although the predominant drug classes varied across regions—with benzodiazepines predominating in a European cohort and antidepressants and antipsychotics in an Asian cohort [9,10,11,21] multiple-pharmaceutical ingestion was a consistent finding, and antidepressant–antipsychotic combinations represented the most common co-ingestion pattern [17]. Multiple-pharmaceutical ingestion may increase the likelihood of clinically significant toxicity through additive pharmacological effects. In keeping with this, severe cases in our cohort were more commonly associated with multidrug overdoses, particularly those involving colchicine or antihypertensive agents, which are well recognized for their potential to cause severe cardiovascular and metabolic toxicity.

Consistent with the previous literature, our cohort showed a marked female predominance [1,2,18]. Large cohorts from Europe and Asia have similarly reported that girls account for approximately 72.5–91.4% of adolescents presenting with intentional pharmaceutical poisoning [9,10,11,15,16,20,22], and our observed proportion of 88.0% falls within this range. The absence of a difference in sex distribution between patients with and without major PICU interventions suggests that this imbalance reflects the higher frequency of intentional self-poisoning among adolescent girls rather than a greater risk of progression to critical illness. This female predominance has been attributed to the higher frequency of non-fatal self-harm behaviors and intentional medication overdoses among adolescent girls, whereas completed suicides remain more common among males because of the use of more violent methods.

Previous cohorts have reported pre-existing psychiatric diagnoses in approximately 60–70.4% of adolescents presenting with intentional pharmaceutical poisoning [9,15], whereas a recent large retrospective study reported a prevalence of 45.8% among younger adolescents, comparable to the 49.6% observed in our cohort [10]. A similar inverse association has been described in previous studies, where adolescents with pre-existing psychiatric diagnoses or prior psychiatric hospitalization tended to present with less severe clinical manifestations despite more frequent self-poisoning episodes, possibly reflecting earlier recognition and closer family supervision [15,17]. One possible explanation is that adolescents without prior psychiatric follow-up may present after less readily recognized or more impulsive self-poisoning episodes, potentially delaying medical evaluation and contributing to greater clinical severity. Nevertheless, the absence of a documented psychiatric diagnosis should not be interpreted as the absence of underlying mental health vulnerability, as many adolescents presenting after self-harm may not yet have undergone formal psychiatric evaluation [15]. This interpretation is further supported by a recent large retrospective cohort in which only 32.8% of adolescents hospitalized after suicide attempts had a previously documented psychiatric disorder, suggesting that self-poisoning may represent the first clinically recognized manifestation of underlying mental health problems in a substantial proportion of adolescents [11]. Accordingly, adolescents presenting with intentional pharmaceutical poisoning should undergo comprehensive psychosocial assessment and timely psychiatric evaluation in addition to acute medical stabilization, consistent with recent evidence and current pediatric recommendations emphasizing integrated mental health assessment, suicide risk screening, and appropriate psychiatric referral [23,24]. Nevertheless, these findings should be interpreted cautiously because our analyses were based on a limited number of outcome events.

A substantial proportion of ingested medications (53.6%) belonged to family members rather than to the patient, and this proportion was significantly higher among patients requiring major PICU interventions (80.0% vs. 50.0%, *p* = 0.026), suggesting that access to household prescription medications may contribute not only to self-poisoning behavior but also to more severe clinical toxicity. Previous studies have likewise identified easy access to prescription medications as an important risk factor for adolescent intentional overdose [1,11,18,22]. Consistent with our findings, Algarrada et al. reported that medications belonging to family members were more frequently involved in moderate-to-severe poisonings, whereas the patient’s own prescriptions showed no such association [15]. In contrast, Wang et al. found that self-poisoning more commonly involved the adolescents’ own prescribed medications among patients receiving psychiatric care [16]. These differences suggest that patterns of medication access may vary according to the characteristics of the study population, particularly the prevalence of pre-existing psychiatric diagnoses and access to personal prescription medications [25].

PSS is a widely used scoring system for standardized assessment of poisoning severity in pediatric toxicology [8]. Although severe or fatal PSS categories were uncommon in our cohort, PSS ≥ 3 was strongly associated with the need for major PICU interventions, supporting the validity of our composite endpoint. Early identification of adolescents at risk of requiring major PICU interventions remains clinically important because significant deterioration may occur despite initially stable vital signs. Comparisons with previous studies should be interpreted cautiously because of differences in severity classification systems and study populations, particularly as emergency department-based cohorts include the full spectrum of poisoning severity, whereas our cohort was limited to patients requiring intensive care admission. Prospective multicenter studies are needed to validate these findings across different PICU admission practices and to develop robust pediatric risk-stratification models for adolescents with intentional pharmaceutical poisoning.

## 5. Limitations

This study has several limitations. First, its retrospective single-center design limits generalizability and introduces the potential for selection bias. Only adolescents admitted to the PICU were included, and admission decisions were individualized according to clinical presentation, anticipated delayed toxicity, characteristics of the ingested pharmaceutical(s), the need for neurological or hemodynamic monitoring, and, when appropriate, recommendations from the National Poison Information Center rather than standardized institutional protocols. Consequently, some patients with an initial Poisoning Severity Score (PSS) of 0 were admitted for close observation because of the risk of delayed deterioration. In addition, our cohort included both direct admissions and inter-hospital transfers, which may have further contributed to selection bias.

Second, toxicological confirmation and accurate information on ingested pharmaceutical doses were unavailable for all patients. Exposure histories relied primarily on patient and family reports, introducing the possibility of recall bias and exposure misclassification.

Third, approximately 28% of patients had missing data on the time from ingestion to hospital admission. Although sensitivity analyses showed no significant differences between patients with complete and missing data, residual bias cannot be excluded.

Finally, the relatively small number of patients requiring major PICU interventions limited multivariable analyses and the precision of subgroup comparisons. Consequently, findings such as the inverse association between psychiatric diagnosis and major PICU interventions should be interpreted cautiously and regarded as hypothesis-generating.

Despite these limitations, this study reflects real-world pediatric intensive care practice and provides clinically relevant data on factors associated with major PICU interventions in adolescents with intentional pharmaceutical poisoning, emphasizing organ-support requirements rather than mortality alone.

## 6. Conclusions

Although most adolescents admitted to the PICU due to intentional pharmaceutical poisoning had a stable clinical course, a clinically important subgroup required major intensive care support. Lower GCS scores, symptomatic presentation, elevated lactate levels, and higher PSS categories were associated with more severe clinical courses and increased intensive care requirements.

Our findings suggest that organ support-based outcomes may better reflect clinically relevant poisoning severity than mortality alone. Furthermore, the high rate of household medication access highlights the importance of safe medication storage and family-based preventive strategies.

## Figures and Tables

**Table 1:** Baseline demographic, psychosocial, clinical, and laboratory characteristics of the cohort.

Variable	Overall Cohort (*n* = 125)
Age, years	16.2 (15.0–16.9)
Female sex	110 (88.0)
Time from ingestion to hospital admission *, h	2.0 (1.0–4.25)
Psychiatric diagnosis	62 (49.6)
Previous suicide attempt	29 (23.2)
Alcohol or recreational substance use	9 (7.2)
Multiple-pharmaceutical ingestion	79 (63.2)
Glasgow Coma Scale	15.0 (15.0–15.0)
pH	7.40 (7.37–7.42)
Lactate, mmol/L	1.20 (0.92–1.54)
Bicarbonate, mmol/L	21.2 (19.6–22.4)
Base excess, mmol/L	−3.3 (−4.4–−1.6)
PICU length of stay, days	2.0 (2.0–3.0)
Mortality	1 (0.8)

Data are presented as median (IQR) or n (%). PICU: Pediatric intensive care unit. * Time from ingestion to hospital admission could be determined in 90 patients.

**Table 2:** Pharmacologic classes involved in ingestion.

Pharmaceutical Class Involved in Ingestion *	Overall Cohort (n = 125)
Antidepressants	46 (36.8)
Analgesics	41 (32.8)
Antipsychotics	39 (31.2)
Antibiotics	10 (8.0)
Methylphenidate	8 (6.4)
Antihypertensive agents	8 (6.4)
Antiepileptic medications	7 (5.6)
Antihistamines	5 (4.0)
Iron preparations	4 (3.2)
Antidiabetic agents	3 (2.4)
Antiarrhythmic agents	3 (2.4)
Colchicine	3 (2.4)

* Patients with multiple-pharmaceutical ingestion were included in more than one pharmaceutical category; therefore, percentages exceed 100%.

**Table 3:** Treatment modalities: Organ support therapies.

Treatment Modality/Outcome	Overall Cohort (*n* = 125)
Gastric lavage	46 (36.8)
Activated charcoal administration	45 (36.0)
Antidote administration	26 (20.8)
Invasive/noninvasive mechanical ventilation	4 (3.2)
Vasoactive support	6 (4.8)
Continuous renal replacement therapy	5 (4.0)
Therapeutic plasma exchange	2 (1.6)
Extracorporeal membrane oxygenation	0 (0.0)

Data are presented as n (%).

**Table 4:** Comparison between patients with and without major PICU interventions.

Variable	No Major PICU Intervention (*n* = 110)	Major PICU Intervention (*n* = 15)	Unadjusted OR (95% CI)	*p* Value
Age, years	16.0 (15.0–16.9)	16.7 (15.8–17.1)		0.356
Female sex	96 (87.3)	14 (93.3)	0.49 (0.06–4.02)	0.434
Time from ingestion to hospital admission *, h	2.0 (1.0–4.75)	2.5 (1.0–7.5)		0.566
Multiple-pharmaceutical ingestion	67 (61.5)	12 (80.0)	2.51 (0.67–9.41)	0.162
Medication belonging to family members	55 (50.0)	12 (80.0)	4.00 (1.07–14.96)	**0.026**
Psychiatric diagnosis	59 (54.6)	3 (20.0)	0.21 (0.06–0.78)	**0.012**
Previous suicide attempt	28 (25.9)	1 (6.7)	0.20 (0.03–1.62)	0.085
Alcohol or recreational substance use	9 (8.4)	0 (0.0)		0.509
PRISM III score > 0	2 (1.8)	3 (20.0)	13.50 (2.05–89.00)	**0.012**
Glasgow Coma Scale < 15	14 (12.7)	5 (33.3)	3.43 (1.02–11.51)	0.053
pH	7.40 (7.37–7.42)	7.38 (7.33–7.42)		0.230
Lactate, mmol/L	1.17 (0.91–1.50)	1.72(1.04–3.80)		**0.010**
Symptomatic presentation	67 (60.9)	14 (93.3)	8.99 (1.14–70.82)	**0.010**
PICU length of stay, days	2.0 (1.0–2.0)	3.0 (3.0–4.0)		**<0.001**
Antidote administration	22 (20.0)	4 (26.7)		0.551
PSS ≥ 3	1 (0.9)	14 (93.3)	Not estimated	**<0.001**

Data are presented as median (IQR) or n (%). Continuous variables were compared using the Mann–Whitney U test. Categorical variables were compared using Pearson’s χ^2^ test or Fisher’s exact test, as appropriate. Major PICU interventions were defined as invasive/noninvasive mechanical ventilation, vasoactive support, continuous renal replacement therapy, therapeutic plasma exchange, ECMO, or severe seizure/arrhythmia requiring intensive care-level management or monitoring. * Time from ingestion to hospital admission could be determined in 90 patients. Bold values indicate statistical significance (*p* < 0.05).

## Data Availability

The datasets generated and/or analyzed during the current study are not publicly available due to privacy and ethical considerations but are available from the corresponding author on reasonable request.

## Supplementary Materials

The following supporting information can be downloaded at https://www.mdpi.com/article/10.3390/children13081058/s1, Table S1. Clinical manifestations on admission and Poisoning Severity Score among patients admitted to the PICU following intentional pharmaceutical poisoning. Table S2. Comparison between patients with asymptomatic and symptomatic poisoning at PICU admission.
