# Supplementary material for: Factors Associated with Major PICU Interventions in Adolescents Hospitalized for Intentional Pharmaceutical Poisoning
Source: Children (Basel). 2026 Aug 8;13(8):1058. doi: 10.3390/children13081058 (PMC13510340; doi:10.3390/children13081058)
Supplement: Supplementary file 1 [file children-13-01058-s001.zip › children-4459368-supplementary.pdf]

Supplementary Tables

Supplementary Table S1. Clinical manifestations on admission and Poisoning Severity Score among patients admitted to the PICU following intentional pharmaceutical poisoning.

Variable	Overall cohort (n=125)
Symptomatic poisoning	81 (64.8)
Nausea/vomiting	42 (33.6)
Dizziness	28 (22.4)
Somnolence/confusion	22 (17.6)
Agitation	14 (11.2)
Hypotension	8 (6.4)
Tachycardia	6 (4.8)
Arrhythmia	5 (4.0)
Seizure	3 (2.4)
Respiratory distress	3 (2.4)
Dystonia	2 (1.6)
Bradycardia	2 (1.6)
Diarrhea	2 (1.6)
Poisoning Severity Score	
0 (none)	45 (36.0)
1 (minor)	54 (43.2)
2 (moderate)	11 (8.8)
3 (severe)	14 (11.2)
4 (fatal)	1 (0.8)

Data are presented as n (%).

Supplementary Table S2. Comparison between patients with asymptomatic and symptomatic poisoning at PICU admission.

Variable	Asymptomatic poisoning (n=44)	Symptomatic poisoning (n=81)	p value
Age, years	16.4 (15.4-16.9)	16.0 (15.0-16.9)	**0.024**
Female sex	38 (86.4)	72 (88.9)	0.441
Multiple-pharmaceutical ingestion	31 (70.5)	48 (60.0)	0.168
Psychiatric diagnosis	27 (61.4)	35 (44.3)	0.052
Previous suicide attempt	11 (25.0)	18 (22.8)	0.473
Psychotropic medication ingestion*	28 (63.6)	41 (50.6)	0.113
Glasgow Coma Scale	15.0 (15.0-15.0)	15.0 (15.0-15.0)	0.091
Lactate, mmol/L	1.22 (0.93-1.52)	1.19 (0.90-1.60)	0.460
pH	7.40 (7.37-7.42)	7.40 (7.36-7.43)	0.917
PSS ≥3	1 (2.3)	14 (17.3)	**0.010**
Major PICU intervention	1 (2.3)	14 (17.3)	**0.010**
PICU length of stay, days	2.0 (1.0-2.0)	2.0 (2.0-3.0)	0.883

Data are presented as median (IQR) or n (%).

*Psychotropic pharmaceutical ingestion included antidepressants, antipsychotics, anxiolytics/sedatives, mood stabilizers, and methylphenidate-containing agents.
